# Multiple-locus variable number of tandem repeat analysis (MLVA) of Irish verocytotoxigenic *Escherichia coli *O157 from feedlot cattle: uncovering strain dissemination routes

**DOI:** 10.1186/1746-6148-4-2

**Published:** 2008-01-24

**Authors:** Mary Murphy, Donal Minihan, James F Buckley, Micheál O'Mahony, Paul Whyte, Séamus Fanning

**Affiliations:** 1 Veterinary Food Safety Laboratory, Cork County Council, Inniscarra, Cork, Ireland; 2 Centres for Food Safety & Food-borne Zoonomics, UCD Veterinary Sciences Centre, University College Dublin, Belfield, Dublin, Ireland; 3 Food Safety Authority of Ireland, Abbey Court, Lower Abbey Street, Dublin 1, Ireland

## Abstract

**Background:**

The identification of the routes of dissemination of *Escherichia coli (E. coli) *O157 through a cohort of cattle is a critical step to control this pathogen at farm level. The aim of this study was to identify potential routes of dissemination of *E. coli *O157 using Multiple-Locus Variable number of tandem repeat Analysis (MLVA).

**Results:**

Thirty-eight environmental and sixteen cattle faecal isolates, which were detected in four adjacent pens over a four-month period were sub-typed. MLVA could separate these isolates into broadly defined clusters consisting of twelve MLVA types. Strain diversity was observed within pens, individual cattle and the environment.

**Conclusion:**

Application of MLVA is a broadly useful and convenient tool when applied to uncover the dissemination of *E. coli *O157 in the environment and in supporting improved on-farm management of this important pathogen. These data identified diverse strain types based on amplification of VNTR markers in each case.

## Background

*Escherichia coli (E. coli) *O157 is a pathogen of serious public health concern. Ruminant animals such as cattle, sheep and goats are asymptomatic carriers of this organism and represent a significant source from where verocytotoxigenic *E. coli *(VTEC) can enter the environment and also the food chain. VTEC can colonise and potentially re-colonise animals for long periods [1]. In cattle the organism has been recognised to colonise the final few centimeters of the gut at the terminal rectum adjacent to the anal canal, from where it coats the faeces as the animal defecates [1]. Although colonisation of the gastrointestinal tract appears to be transient [2], persistent shedders of high quantities of these organisms can exist within groups of cattle [3,4]. This can lead to a rapid transmission of *E. coli *O157 amongst housed cattle, with contamination of the drinking water, pen barriers and hides possibly occurring within twenty-four hours [2].

The bacterium survives in the farm environment. On-farm sources from which these *E. coli *O157 organisms have been isolated include water, water troughs, stored animal faeces and feed. Studies have shown that *E. coli *O157:H7 can survive in animal faeces for up to 97 days and 109 days in water [5]. Water troughs on farms have been frequently found to contain VTEC, which could in turn act as a source from which the bacterium can spread to large numbers of animals over a short period of time [6,7]. Farmyard gates and stiles also pose a direct risk as *E. coli *O157 have the potential to persist for long periods on these surfaces [8]. Contamination of the environment by *E. coli *O157 is also a serious threat to those working on farms (including animal transporters) and also those visiting farms especially young children [9].

Recently, Multi-Locus Variable Number of Tandem Repeat Analysis (MLVA) has become an established technique to subtype *E. coli *O157 [10-14]. MLVA is a PCR-based subtyping method that can be used to discriminate amongst different strains of a bacterium based on differences in the number of tandem repeats (TRs). The number of TRs at a specific locus in the genome of a microorganism can vary as a consequence of DNA polymerase enzyme slippage during replication. Detection of these TR differences can be achieved by PCR incorporating primer pairs designed to anneal to the flanking regions of each TR followed by amplification and conventional agarose gel electrophoresis [15].

In this paper we describe the application of MLVA sub-typing to uncover the dynamics of strain dissemination in a cattle feedlot.

## Results

Locus-specific PCR primer sets previously characterised by Noller *et al*., (2003) [12] were used to amplify each of seven TR regions using single primer-paired reactions for all fifty-four isolates analysed. Amplified products were produced for the majority of the *E. coli *O157 strains with the following exceptions; isolates CFSE-2, -3, -4, -16, -23 and -50 (Table 1) did not produce any amplicon for the TR-1 locus, similarly CFSE-2 for TR-2, CFSE-16 for TR-3, CFSE-23 for TR-5 and CFSE-28 for the TR6, amplicon. The numbers of repeats identified in this collection, ranged from 0 to 18 across the seven loci analysed and these are presented in Table 1. Diversity indices were calculated for all TRs using Simpson's index with values ranging between 55.9 and 92.2% (Table 2). The highest level of diversity (92.2%) was among the repeats identified at TR-2.

**Table 1:** Characteristics of the study collection of *Escherichia coli *O157 isolates.

**Isolate Number**	**Month of Isolation**	**Pen No.**	**Sample Type**	**No. of repeats**							**MLVA Type**
					
				**TR1**	**TR2**	**TR3**	**TR4**	**TR5**	**TR6**	**TR7**	
CFSE-23	January	2 & 3	Pen bars	np	04	07	00	np	00	05	1
CFSE-24	January	2 & 3	Pen bars	05	03	07	00	03	00	05	1
CFSE-8	January	3	Pen floor faeces	05	05	07	00	03	00	05	1
											
CFSE-25	January	3 & 4	Water trough sediment	05	03	06	00	03	00	05	1
CFSE-19	February		Slurry tank faeces	05	08	07	01	02	00	04	2
CFSE-18	February		Slurry tank faeces	06	09	06	01	02	00	04	2
											
CFSE-20	December	1 & 2	Pen bars	08	08	07	00	02	00	06	3
CFSE-21	December	2 & 3	Pen bars	08	08	07	00	02	00	06	3
CFSE-22	January	1 & 2	Pen bars	08	08	07	00	02	00	05	3
											
CFSE-55	November	2	Cattle faeces	08	15	04	01	03	00	06	4
CFSE-56	November	2	Cattle faeces	08	14	04	01	03	00	06	4
CFSE-53	December	1	Cattle faeces	08	14	06	01	03	00	06	4
CFSE-52	December	1	Cattle faeces	08	12	07	01	03	00	06	4
CFSE-7	January	1	Pen floor faeces	08	11	06	00	04	00	06	4
											
CFSE-47	November	4	Cattle faeces	06	06	08	02	03	00	05	5
CFSE-48	November	4	Cattle faeces	06	07	08	02	04	00	05	5
CFSE-46	December	4	Dust	06	07	07	03	04	01	05	5
CFSE-49	December	4	Cattle faeces	06	05	09	02	04	00	04	5
CFSE-50	December	4	Cattle faeces	np	06	08	03	04	00	05	5
											
CFSE-5	December	4	Pen floor faeces	06	08	06	01	03	01	05	6
CFSE-6	December	4	Pen floor faeces	05	07	06	01	04	01	05	6
CFSE-44	December	2	Feed	06	12	06	02	04	01	06	6
CFSE-59	January	3	Cattle faeces	06	06	06	02	03	01	06	6
CFSE-60	January	3	Cattle faeces	06	06	06	01	03	01	06	6
CFSE-61	January	3	Cattle faeces	06	07	06	02	03	01	06	6
CFSE-62	January	3	Cattle faeces	06	07	07	02	03	01	07	6
											
CFSE-34	November	2	Feed trough swab	06	13	06	02	03	00	05	7
CFSE-35	November	2	Feed trough swab	07	12	05	02	03	00	04	7
CFSE-36	December	1	Feed trough swab	07	12	06	02	03	00	04	7
CFSE-37	December	2	Feed trough swab	06	11	05	02	03	00	06	7
CFSE-17	January		Slurry tank faeces	06	10	07	02	03	00	05	7
											
CFSE-39	December	4	Feed trough swab	08	05	05	02	03	00	05	8
CFSE-54	December	1	Cattle Faeces	08	10	05	01	03	00	05	8
CFSE-38	December	3	Feed trough Swab	05	10	05	02	03	00	05	8
CFSE-40	January	3	Feed trough swab	05	05	05	02	03	00	05	8
CFSE-41	January	4	Feed trough swab	05	05	05	02	03	00	05	8
											
CFSE-57	November	2	Cattle faeces	08	17	05	02	04	01	07	9
CFSE-58	November	2	Cattle faeces	08	17	05	02	03	01	07	9
CFSE-51	December	1	Cattle faeces	08	18	08	02	04	00	08	9
CFSE-45	January	1	Feed	09	12	07	02	04	01	07	9
											
CFSE-28	December	1 & 2	Agitated water	09	11	08	01	04	np	07	10
CFSE-9	February	2	Pen floor faeces	09	08	07	01	04	00	06	10
CFSE-10	February	2	Pen floor faeces	09	08	08	01	04	01	07	10
											
CFSE-32	January	1 & 2	Water trough swab	07	08	07	02	05	00	06	11
CFSE-30	February	3 & 4	Agitated water	07	11	08	02	05	01	06	11
CFSE-13	February	3	Pen floor faeces	08	08	08	02	05	02	06	11
CFSE-14	February	4	Pen floor faeces	08	05	08	01	05	02	06	11
CFSE-15	February	4	Pen floor faeces	08	04	08	02	05	01	06	11
											
CFSE-1	November	2	Pen floor faeces	09	07	06	03	04	02	06	12
CFSE-2	November	3	Pen floor faeces	np	np	06	03	04	02	05	12
CFSE-3	December	2	Pen floor faeces	np	14	06	03	04	02	05	12
CFSE-4	December	2	Pen floor faeces	np	14	06	02	04	01	05	12
CFSE-16	December		Slurry tank faeces	np	11	np	02	04	02	07	12
CFSE-43	February	3	Feed trough swab	09	06	06	02	04	01	05	12

np, denotes the absence of PCR product at the corresponding locus.

**Table 2:** Primer characteristics [12] and calculated % diversity indices for each of the tandem repeats (TRs).

**Primer**	**Sequence 5' to 3'**	**Size [mer]**	**Tm °C**	**TR Sequence**	**TR size**	*Diversity Index (%)
TR 1	-ACT GCA TGA TAA GCC TCA GG-	20				
	-CAC TGA AGC CTG TTC CGT TC-	20	57	-AAATAG-	6	77.0
TR 2	-CGC AGT TGA TAC CTA CGG -	18				
	-GGA AGG AAG CTG ATA GGT -	18	53	-TGGCTC-	6	92.2
TR 3	-TCT TGT CAA TAT AGA TTG G-	19				
	-TGA TTA AGC GTG TAC TGA -	18	50	-TATCTT-	6	77.6
TR 4	-GGT GAT GGC TTG ATA TTG A-	19				
	-GCC ACA CTG CGA GTA TAG AG-	20	53	-TGCAAA -	6	66.5
TR 5	-GTT GAT TAT CAT GGT ATG TC-	20				
	-GGA CAA CTT GTA GTA CAA G-	19	51	-AAGGTG -	6	66.1
TR 6	-GAT GGT TCG ACT AAC CGT TAT-	21				
	-TAG CAG ATG TTC GTT CCT-	18	53	-TTAAATAATCTACAGAAG -	18	55.9
TR 7	-CGC AGT GAT CAT TAT TAG C-	19				
	-TGC TGA AAC TGA CGA CCA GT-	20	50	-GACCAC-	6	71.3

* Diversity is based on Simpson's index calculated using all fifty-four isolates

**Figure 1:**
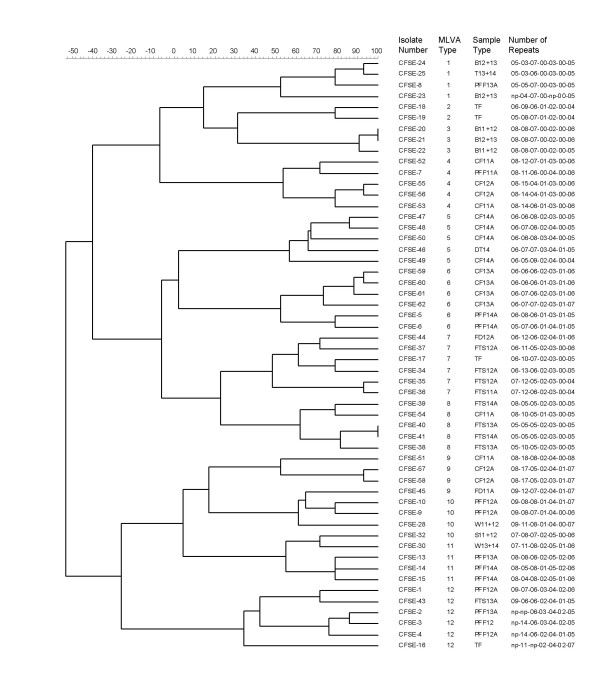
**Dendrogram**. A dendrogram of the Multi-locus variable number of tandem repeat analysis (MLVA) of the fifty-four *Escherichia coli *O157 isolates. The scale shown represents % similarity.

**Figure 2:**
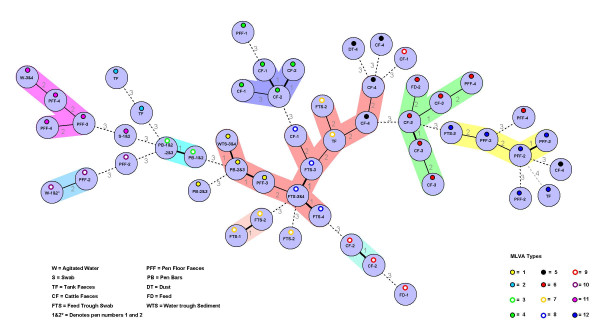
**Minimum Spanning Tree**. Minimum spanning tree of the Multi-locus variable number of tandem repeat analysis (MLVA) of the fifty-four *Escherichia coli *O157 isolates. The numbering shown within each circle denotes the pen number from where each isolate was recovered. Each isolate is colour coded according to their corresponding MLVA type. The colour coding is identified by the key accompanying this figure (see also Table 1). The numbering shown between the circles represents the distance between each node.

Based on the MLVA types defined above, a dendrogram was created to cluster the isolates (Figure 1). Analysis of these repeats identified twelve MLVA profiles (Table 1). A minimum spanning tree (MST) was also constructed which was rooted to the isolate with the highest number of related isolates to it [i.e. isolates CFSE-40 and -41 cultured from feed trough swabs in pens 3 and 4 in January, (FTS-3 and 4, MLVA type-8, Figure 2)]. All other isolates were derived from this root demonstrating the overall genetic clustering that existed amongst these strains. A number of distinct clusters were noted in the MST and as expected the main clustering occurred within pens, in cattle faecal isolates and also environmental samples from respective pens.

**Figure 3:**
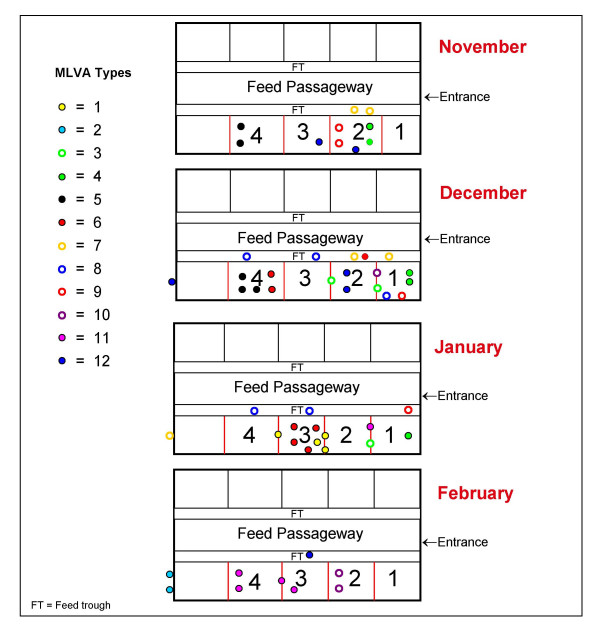
**Feedlot Layout**. Schematic representation of the spatial and chronological distribution of *Escherichia coli *O157 clusters in this feedlot (see Figure 1). Coloured dots represent the Multi-locus variable number of tandem repeat analysis (MLVA) types. Red lines between pens indicate pen bars and dots on lines indicate the MLVA type exhibited by isolates detected on pen bars or in water troughs between pens. Coloured dots outside the feedlot represent the MLVA types displayed by those strains cultured from slurry tank faecal samples.

A schematic representation of the spatial and chronological distribution of *E. coli *O157 clusters in this feedlot is shown in Figure 3. The pen floor layout highlights the corresponding MLVA types of each strain over the four-month study period. Multiple *E. coli *O157 strains were detected in each pen as indicated by the MLVA types (Figures 1, 2 and 3). Strains of *E. coli *O157 with MLVA type 4 were cultured from cattle faecal samples in pen 2 in November. By December, strains with this particular type were cultured from cattle faecal samples in pen 1, highlighting possible transfer of this strain between pens (MLVA type 4, Table 1; Figures 1, 2 and 3).

Strains of MLVA type 5 were cultured from cattle faecal samples in pen 4 in November and again in December (MLVA type 5, Table 1; Figures 1, 2 and 3). *E. coli *O157 strains with MLVA type 6 were cultured in December from faeces from the floor of pen 4 and in a feed sample in pen 2. By January this MLVA type was detected in the four cattle faecal isolates in pen 3, showing a probable horizontal transmission route through feed/feed troughs (MLVA type 6, Table 1; Figures 1, 2 and 3).

Strains of MLVA type 7 were cultured in feed trough swab samples in pen 2 in November and by December strains with this type were detected in feed trough swab samples in pens 1 and 2 (MLVA type 7, Table 1; Figures 1, 2 and 3). Isolates from the dividing bars located between pens 1 and 2 and again between pens 2 and 3, in December, included strains with indistinguishable MLVA types. A similar profile was observed in isolates obtained from the same sites in January (MLVA type 3, Table 1; Figures 1, 2 and 3), suggesting horizontal transmission between adjacent pens.

Strains of MLVA type 9 were cultured in cattle faecal samples in pen 2 in November and by December this strain was cultured from cattle faecal samples in pen 1 and in feed samples cultured in pen 1 in January (MLVA type 9, Table 1; Figures 1, 2 and 3).

Strains of MLVA type 11 were cultured from water trough swab samples in January and by February strains with this profile were cultured from pen floor faeces in pens 3 and 4, and the agitated water in the water trough located between pens 3 and 4, highlighting water as a possible transmission source of this pathogen (MLVA type 11, Table 1; Figures 1, 2 and 3). The *E. coli *O157 strains displaying MLVA type 2 were only cultured from slurry tank faeces.

## Discussion

Our data show that, the application of MLVA to broadly discriminate between different strains of *E. coli *O157 may be a useful tool to aid in detecting strain dissemination patterns. MLVA could separate isolates into clusters dividing the fifty-four isolates into twelve different MLVA types. As no *E. coli *O157 was detected from environmental samples prior to restocking, these data support the conclusions of Minihan et al., (2003) [16] that animals entering the feedlot and shedding *E. coli *O157 at initial stocking, are an important source of contamination. These animals are probably responsible for the initial inoculation of the immediate environment. Once *E. coli *O157 inoculated the feedlot, water troughs, pen bars, pen floor faeces and feed all became important sources for transmission of the organism.

Application of a MLVA typing scheme in a veterinary setting presents unique challenges. Choice of the TR loci for the analysis is an important step in the success of any subtyping protocol. As several loci specific to *E. coli *O157 were previously characterised by Noller *et al*., (2003) [12], these markers were included in this study. These TR loci along with others have been previously applied in other studies, for example, four of the seven loci were included by Lindstedt *et al*., (2003) [11] in their study and all seven loci were included in a study by Keys *et al*., (2005) [14]. Other TR markers have been defined more recently [17].

Based on our data, we calculated diversity indices for the seven TR loci with values ranging from 55.9 to 92.2% (Table 2). The highest level of diversity observed (92.2%) related to TR-2 and this was the most polymorphic locus observed in our study. Similar observations were reported earlier by Noller *et al*., (2003 and 2006) [12,18], Keys *et al*., (2005) [14] and by Kawamori *et al*., (2008) [17]. It was previously suggested by Keys *et al*., (2005) [14] that markers with high diversities are crucial in discriminating between closely related isolates. Keys *et al*., [14] highlighted the importance of including TR-2 (O157-10), as an example of a crucial marker.

Careful interpretation of data obtained from the analysis of highly discriminating loci, in closely related strains and detecting strain differences between distantly related strains is important in sub-typing. Mutational rates differ within tandem repeat loci thereby increasing the polymorphism associated with these markers. Noller *et al*., (2006) [18], defined isolates as belonging to the same lineage when no more than a single VNTR difference occurred between all loci. Based on our VNTR data, TR-2 was highly polymorphic and few of our isolates conformed to Nollers' definition.

Interestingly, the majority of the strains examined in this study had no repeats at the TR6 locus and were designated as "00" whilst the remaining strains contained just one or two repeats as indicated by the identifiers "01" or "02" respectively (Table 1). Comparing these data to those reported earlier by Noller *et al*., (2003) [12], highlighted differences in repeat number (at this locus) which may (at least in part) reflect the origin of our strain collection, compared to others (using the same set of TR loci). Nonetheless the MST generated based on these data, facilitated the localised tracing of routes of dissemination in the pens, as illustrated in Figure 3.

The environment of the feedlot was highlighted as an important source of transmission of *E. coli *O157 when a single strain was detected in the pen floor faeces in pen 3, sediment samples from the water trough located between pens 3 and 4 and from the pen bar barriers between pens 2 and 3 in January (MLVA type-1). Once contaminated the environment had the potential to act as a reservoir of infection/re-infection not only for cattle, but also for other possible vectors including flies and rodents [19].

In this study, *E. coli *O157 was detected in both feed and water troughs, which is consistent with previous reports [7,20]. On many farms, water troughs are infrequently cleaned which gives rise eventually to the development of thick sediments accumulating, along with biofilm formation. These may then serve as a long-term source of pathogen infection of bovine and other animals [7]. As cattle in two or more feedlot pens may drink from communal water troughs, our study highlighted the importance of having drinking systems in place, designed to minimize the entry of faecal material. In feedlot design, consideration should be given to the incorporation of unique water trough access to each pen. Feed troughs should also be designed in such a way that they are not amenable to faecal contamination, and inter-pen transmission. Attention should also be paid to the risks posed by human traffic moving between pens within a feedlot environment.

The application of MLVA is a useful tool in broadly establishing the dissemination routes of *E. coli *O157 in the environment. This sub-typing protocol facilitated the description of the dissemination patterns among a collection of isolates and the identification of important risk factors. Choice of the repeat loci to include in the analysis is important, as highlighted by the findings of this and other studies. Noller *et al*., (2006) [18] recently put forward suggestions for the interpretation of MLVA data. In the future loci used to investigate animal and human populations of *E. coli *O157 should be standardised, facilitating an improved understanding of the dissemination of this human pathogen through the food chain. Nevertheless, MLVA is a technique that could be applied by most laboratories and as we have shown, these modern laboratory tools can usefully support on-farm management practices aimed at reducing the transmission of this important human pathogen, early in the food chain.

## Conclusion

Through the use of MLVA, our study demonstrates some of the transmission routes of *E. coli *O157, cultured from bovine animals. Strain diversity was observed within pens, individual animals and their environment. The overlap between pens suggests dissemination of the bacterium within the feedlot environment with possible involvement of selection pressures for those specific strains in the same environment. Our data also suggested that cohorts of cattle can be exposed to and become asymptomatic carriers of a number of *E. coli *O157 strains differentiated according to their MLVA profiles [21,22]. Results obtained provided an insight into the longitudinal dynamics of *E. coli *O157 dissemination and identified dominant strains emerging and re-emerging over time [23,24]. The implementation of good hygiene practices along with other biosecurity measures linked to MLVA profiling may help to control the dissemination of *E. coli *O157 within a feedlot environment and contribute to improvements in HACCP-based management plans, to protect public health.

## Methods

### Bacterial strains

The isolates used in this investigation were obtained from a previous study [16] wherein monthly environmental and rectal faecal samples were collected from 133 heifers and the four adjacent holding pens that they occupied in a freshly populated feedlot over a five-month period (November to March). No *E. coli *O157 was isolated from a range of environmental samples taken in the feedlot prior to it's restocking. The environmental samples investigated in this study included faeces from the pen floors and slurry tank, dust, water and sediment from the water troughs, feed and swabs from the feed troughs and pen separating bars. All *E. coli *O157 isolates were detected and identified previously [16] using immunomagnetic separation and all isolates were maintained at -80°C on cryostat beads. Fifty-four *E. coli *O157 isolates (composed of 38 environmental and 16 animal isolates cultured between November and February) were selected for this study from a total of 182 isolates recovered (46 environmental isolates and 136 animal isolates).

### Template DNA purification

Briefly, all bacteria were resuscitated by culture on Columbia blood agar [Oxoid, Basingstoke, U.K.] at 37°C for 16 h and a single isolated colony was inoculated into 3 ml Tryptone-Soy-Broth (TSB), [Oxoid] and incubated at 37°C overnight. The overnight culture was recovered by centrifugation at 13,000–16,000 × g for 2 min. The supernatant was discarded and total genomic DNA was purified using the Promega Wizard Genomic DNA purification kit (Promega, Madison, WI) according to the manufacturers' recommendations for Gram-negative bacteria.

### Multiple-Locus Variable number of tandem repeat Analysis (MLVA)

Primer sets reported previously [12], (Table 2) were used to amplify the selected genomic regions known to contain the tandem repeat (TR) structures, from all fifty-four isolates. Amplification of TR sequences from purified bacterial genomic DNA [approx. 100 ng] was performed in 20 μl volumes each containing 1 × PCR buffer (750 mM Tris-HCl, [pH 8.8], 200 mM (NH_4_)_2_SO_4_, 0.1% (v/v) Tween 20), 2 mM MgCl_2_, 200 μM of each dNTP, 0.5 μM corresponding primers along with 0.5 U Red Hot *Taq *DNA polymerase (Abgene, Surrey, U.K). Thermal cycling consisted of 35 cycles, of 94°C for 45 s, annealing temperature was set according to the T_m _values shown (in Table 2) for 45 s followed by 72°C for 60 s, with a final extension at 72°C for 5 min., (a modification of the cycling conditions by Noller *et al*., 2003 [12]). Each amplification reaction included a positive control and a no-DNA template control. All amplifications were performed using a thermal cycler PTC-200 DNA Engine, (MJ Research Inc., Waterton, MA). Amplified products (5 μl volumes) were resolved by conventional electrophoresis through horizontal 2% (w/v) agarose gels at 100 V for approximately 200 min., in 1 × TBE buffer (89 mM Tris base, 89 mM Boric acid, 0.5 M EDTA [pH 8.0]) containing 0.5 μg/ml ethidium bromide and the results visualised and photographed in a Gel Doc 2000 system (BioRad, Hercules, CA). An equal concentration of two molecular weight markers (VIII, Roche Applied Science, Mannheim, Germany and 100 bp DNA Ladder, New England Biolabs, Inc., Ipswich, MA), were included in all gels to facilitate the sizing of amplified DNA fragments. Once each amplicon was sized the number of TRs was calculated using Quantity One software (Biorad, Hercules, CA). An allele number string, based on the number of TRs at each locus was assigned to the amplified DNA fragments. The number of TRs was rounded down to an integer value.

### Data Analysis

All of the allele strings were imported into a Bionumerics software package (version 4.5; Applied Maths, Sint-Martems-Latem, Belgium) and a minimum spanning tree was created based on categorical and the priority rule highest number of single locus variants (SLV's). In cases where two types had an equal distance to a linkage position in the tree, the type with the highest number of SLVs (i.e. other types that differ only in one state or character) were linked first. The genetic diversity at each TR was calculated using Bionumerics software package using Simpson's Index. A dendrogram was also constructed according to the Ward algorithm, using the multistate categorical coefficient with a tolerance level of two and enabling fuzzy logic.

## Authors' contributions

MM was responsible for carrying out this study and was the main contributor to writing the manuscript. DM carried out the original epidemiological study. JB and MO'M provided veterinary expertise for the study. PW and SF co-ordinated the project and reviewed all drafts of the manuscript. All authors read, commented on and approved the final manuscript.
